# Initiating Binary Metal Oxides Microcubes Electromagnetic Wave Absorber Toward Ultrabroad Absorption Bandwidth Through Interfacial and Defects Modulation

**DOI:** 10.1007/s40820-023-01197-0

**Published:** 2023-10-09

**Authors:** Fushan Li, Nannan Wu, Hideo Kimura, Yuan Wang, Ben Bin Xu, Ding Wang, Yifan Li, Hassan Algadi, Zhanhu Guo, Wei Du, Chuanxin Hou

**Affiliations:** 1 School of Environmental and Material Engineering, Yantai University, No. 30 Qingquan Road, Yantai, Shandong 264005 People’s Republic of China; 2 School of Material Science and Engineering, Shandong University of Science and Technology, Qingdao, 266590 People’s Republic of China; 3 Mechanical and Construction Engineering, Faculty of Engineering and Environment, Northumbria University, Newcastle Upon Tyne, NE1 8ST UK; 4 College of Materials Science and Engineering, Taiyuan University of Science and Technology, Taiyuan, 030024 People’s Republic of China; 5 Department of Electrical Engineering, Faculty of Engineering, Najran University, 11001 Najran, Saudi Arabia

**Keywords:** Electromagnetic wave absorber, NiCo_2_O_4_@C Microcubes, Oxygen vacancy, Effective absorption bandwidth

## Abstract

Cobalt nickel bimetallic oxides (NiCo_2_O_4_) have received numerous attentions in terms of their controllable morphology, high temperature, corrosion resistance and strong electromagnetic wave (EMW) absorption capability. However, broadening the absorption bandwidth is still a huge challenge for NiCo_2_O_4_-based absorbers. Herein, the unique NiCo_2_O_4_@C core–shell microcubes with hollow structures were fabricated via a facile sacrificial template strategy. The concentration of oxygen vacancies and morphologies of the three-dimensional (3D) cubic hollow core–shell NiCo_2_O_4_@C framework were effectively optimized by adjusting the calcination temperature. The specially designed 3D framework structure facilitated the multiple reflections of incident electromagnetic waves and provided rich interfaces between multiple components, generating significant interfacial polarization losses. Dipole polarizations induced by oxygen vacancies could further enhance the attenuation ability for the incident EM waves. The optimized NiCo_2_O_4_@C hollow microcubes exhibit superior EMW absorption capability with minimum RL (*RL*_min_) of −84.45 dB at 8.4 GHz for the thickness of 3.0 mm. Moreover, ultrabroad effective absorption bandwidth (EAB) as large as 12.48 GHz (5.52–18 GHz) is obtained. This work is believed to illuminate the path to synthesis of high-performance cobalt nickel bimetallic oxides for EMW absorbers with excellent EMW absorption capability, especially in broadening effective absorption bandwidth.

**Graphic Abstract:**

**Supplementary Information:**

The online version contains supplementary material available at 10.1007/s40820-023-01197-0.

## Highlights

- 3D cubic hollow core–shell NiCo_2_O_4_@C composites were synthesized.
- Oxygen vacancies were introduced into the prepared composites.
- Ultrabroad effective absorption bandwidth of 12.48 GHz was obtained.
- The absorption performance mechanism of NiCo_2_O_4_/C composites was investigated.

**Supplementary Information:**

The online version contains supplementary material available at 10.1007/s40820-023-01197-0.

## Graphical abstract

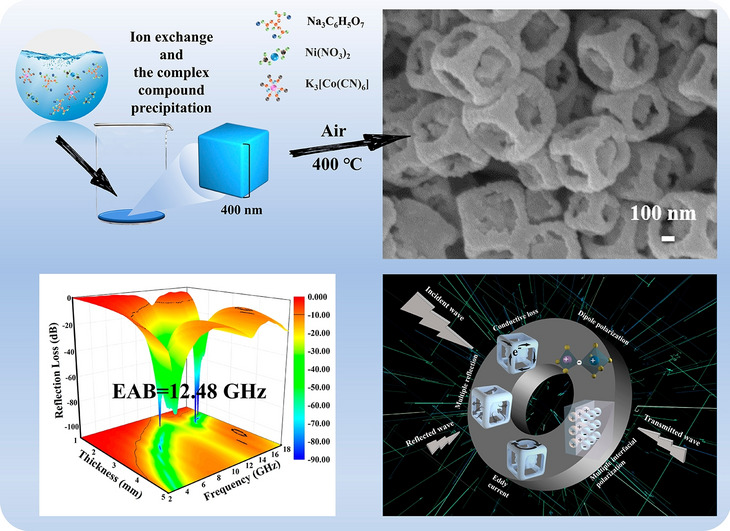

## Introduction

The emergence of various wireless electronic devices was promoted by the rapid development of science and technology, provides people with convenience. However, the adverse effects of the operation and maintenance of sophisticated instruments, human organ damage, and even the survival of certain organisms were also produced by the electromagnetic radiation, which was called electromagnetic pollution [1–3]. For the sake of dealing with electromagnetic pollution, EMW absorbers have received numerous attentions due to their ability to weaken or/and dissipate EMW by converting them into other forms of energy [4–6]. The perfect electromagnetic wave absorber should satisfy the characteristics of strong absorption intensity, broad absorption bandwidth, thin matching thickness, and light weight. In the past decades, various materials have been carried out in EM absorption field [7–11], such as magnetic loss materials (*e.g.,* ferrite, metal alloys) and dielectric loss materials (*e.g.*, carbon-based materials, conductive polymers, and ceramics).

Among the plentiful EMW absorber materials, bimetallic cobalt–nickel oxide materials are receiving immense attentions due to their low synthesis cost, controlled morphology, and environmental friendliness. However, pure nickel cobaltate nanoparticles and nanosheets suffered from relatively poor EMW absorption ability with an *RL*_min_ value of only −11 dB. The EMW absorption performance of pure nickel cobaltate nanoparticles is restricted due to impedance mismatch, which was caused by the poor magnetic loss capability [12].

To optimize the impedance matching to acquire satisfactory cobalt nickel bimetallic oxides absorber, two conventional strategies has been proposed. Designing and preparation of unique structure, including hollow porous structures, core–shell structures, etc., is considered as one effective route [13]. For example, the NiCo_2_O_4_ with porous hollow spherical structure and additional flower-like and flower-sphere structures were obtained by changing the type of surfactant, which exhibited an *RL*_min_ value of −31.1 dB and an EAB value of 5.44 GHz at the thickness of 1.8 mm [14]. The uniquely designed structure with abundant microporous channels was proved to facilitate the multiple reflections and scatterings of incident EMW. Fabricating composites with multiple interfaces is believed to be another strategy to promote the EMW absorption capacity by improving impedance matching and interface polarization ability simultaneously. Recently, defects-induced polarizations have been proved to improve the dielectric loss capacity effectively [15]. For example, Co_3_O_4_@NiCo_2_O_4_ composites were synthesized and exhibited an EAB value of 4.88 GHz at 2.6 mm [14]. Hierarchical C/NiCo_2_O_4_/ZnO composites were fabricated by hydrothermal, and sol–gel method, which delivered an EAB value of 4.32 GHz at a thickness of 2.4 mm [13, 16]. The existence of multi-component is conducive to inducing interface polarization, enhancing the dielectric loss ability [17].

Besides the two conventional strategies, defect engineering is also an effective tactic to improve the EMW absorption performance. Defect locations such as vacancies could trap carriers and disrupt the balance of charge distribution, resulting in defect polarization and loss of electromagnetic energy. NiCo_2_O_4_ absorbers with rich oxygen vacancies and functional groups were fabricated, and their EAB could cover the entire Ku-band [18]. Although extensive research have been carried out on NiCo_2_O_4_ absorber and achieved exceptional absorption performance [19], the demands for NiCo_2_O_4_-based EMW absorbers with broadened absorption bandwidth are still a huge challenge.

Herein, the three-dimensional NiCo_2_O_4_@C hollow core–shell microcubes with rich oxygen vacancy defects were synthesized via facile chemical precipitation and following the calcination process. This special structure is forecast to provide multi-advantages to boost the EMW absorption performance. First, the special cubic hollow core–shell framework facilitates the entry of EMW and multiple reflections. Second, the introducing of carbon during the synthesis process facilitates the induction of interfacial polarization and reduces the density of the material to achieve a lightweight effect. Besides, the oxygen vacancy defects act as a dipole to induce dipole polarization and effectively enhance the dielectric loss capacity of the absorber. In terms of the synergistic effect of hollow microstructure, multi-component, and defect engineering, the optimized NiCo_2_O_4_@C absorber showed an *RL*_min_ value of −84.45 dB and ultra-broad EAB value as large as 12.48 GHz (5.52–18 GHz), which is the largest among the current reports. This work tremendously broadens the effective absorption bandwidth of NiCo_2_O_4_-based absorbers, which will accelerate its practical application in EM absorption fields.

## Experimental Section

### Materials

Sodium citrate (C_6_H_5_O_7_Na_3_, ≥ 98.0%), Nickel nitrate (Ni(NO_3_)_2_∙6H_2_O, ≥ 99.0%), and Potassium hexacyanocobaltate (III) (C_6_CoK_3_N_6_, ≥ 99.9%) were provided by Rgent, Dingshengxin and Macklin, respectively. All analytical reagents were used directly without further purification.

### Synthesis of NiCo_2_O_4_@C Composites

The Ni–Co–PBA nanocubes were obtained by a typical precipitation method. 2.4 mmol C_6_H_5_O_7_Na_3_ and 1.6 mmol Ni(NO_3_)_2_∙6H_2_O were dissolved into 60 mL of deionized (DI) water, stirring for 10 min to form uniform solution I. Meanwhile, 1.0 mmol C_6_CoK_3_N_6_ was dissolved into 40 mL of DI water to form uniform solution II. Then, the solution I and II were mixed under stirring for 10 min. The acquired mixed solution was kept for 12 h at room temperature. The precipitation products were collected by centrifugation and washing with water and ethanol several times and dried at 60 °C for 12 h. The final products were obtained after heat treatment of Ni–Co–PBA nanocubes precursor at 360, 380, 400 and 420 °C in air for 2 h with a heating rate of 2 °C min^−1^, which marked as NCO-1, NCO-2, NCO-3 and NCO-4, respectively.

### Characterization

The phase structure was characterized by X-ray diffraction (XRD) using a Rigaku D/max-3C. The morphologies and microstructures were analyzed by a scanning electron microscope (SEM, JSM-7610F, Japan) and a transmission electron microscope (TEM, TF20), respectively. The composition and valence of elements of the prepared NiCo_2_O_4_ nanomaterials were determined by X-ray photoelectron spectrometer (XPS, Thermo Scientific K-Alpha), Raman spectrometer (Horiba LabRAM HR Evolution), and TGA/DSC system (Netzsch STA 449 F3). The magnetic characters of the as-prepared samples were observed via a vibrating sample magnetometer (VSM, LakeShore7404, USA) at room temperature. The specific surface and pore size distribution were analyzed by nitrogen adsorption–desorption isotherms (ASAP 2460). To demonstrate the presence of oxygen vacancies, the samples were tested using electron paramagnetic resonance spectroscopy (EPR, Bruker EMXplus-6/1, Germany).

### Electromagnetic Measurements

To evaluate the electromagnetic parameters, a vector network analyzer (3656D) was utilized between 2 and 18 GHz. An appropriate amount of the prepared sample and paraffin was mixed by heating and stirring, and transferred into in the mold to form a test ring (Φouter = 7.00 mm, Φinter = 3.04 mm, thickness = 2.0 mm) with a filling ratio of 20, 30, and 40%.

## Results and Discussion

**Fig. 1:**
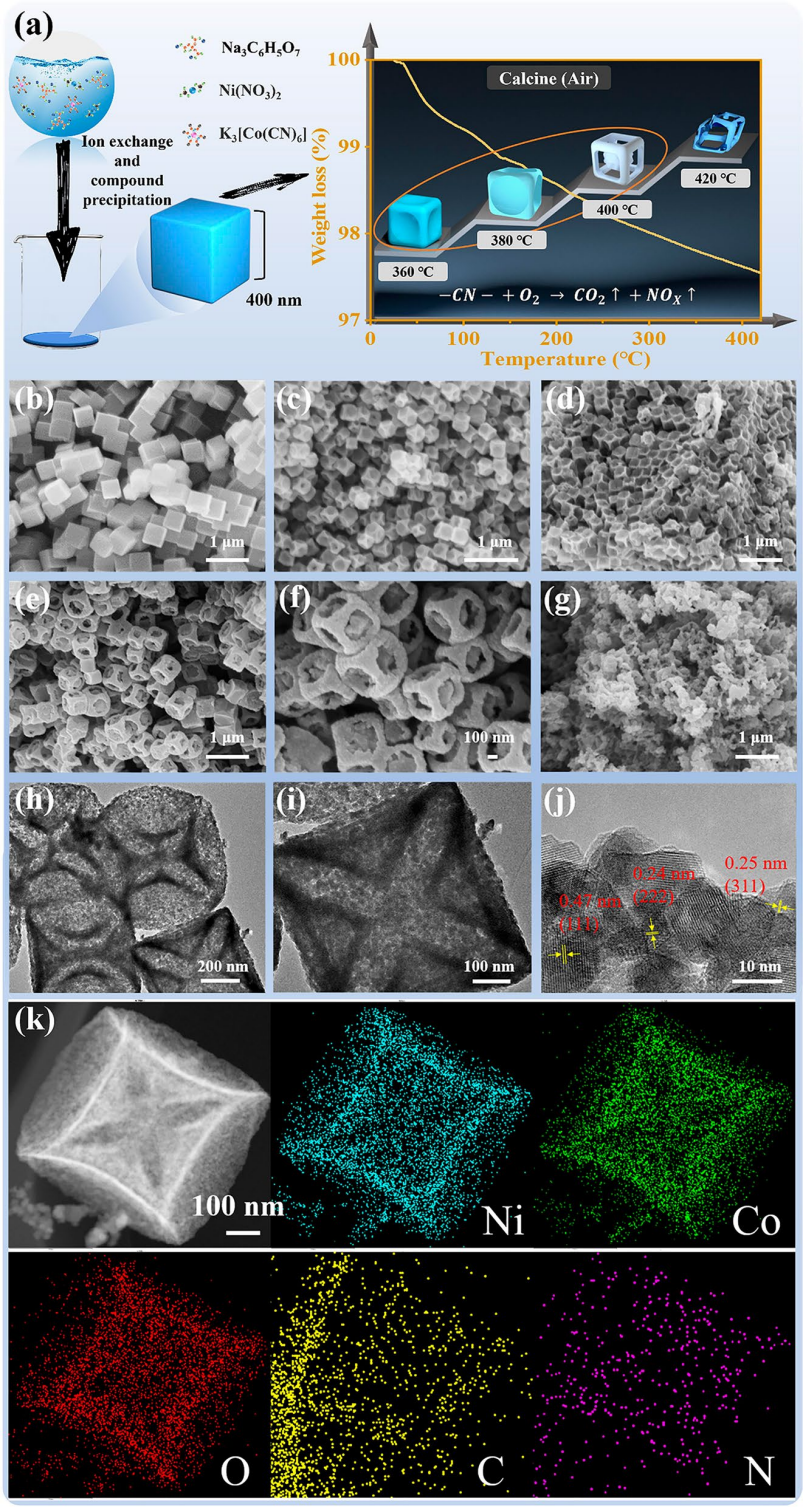
**a** Schematic illustration of the preparation route of the 3D hollow NiCo_2_O_4_ microcubes. SEM images of **b** Ni–Co–PBA, **c** NCO-1, **d** NCO-2, **e–f** NCO-3 and **g** NCO-4; **h–j** TEM images and **k** TEM-based EDS mapping of NCO-3

The synthesis route of the 3D cubic hollow core–shell NiCo_2_O_4_@C framework composites with oxygen vacancy defects is schematically described in Fig. 1a. The dissociated Ni^2+^ coordinated with [Co(CN)_6_]^3−^ to produceg large number of Ni–Co–PBA nanoclusters, which aggregated into primary nanoparticles at supersaturations by van der Waals forces in an oriented manner. During this process, citrate will adsorb on the surface of the nanoparticles to control the binding rate. The morphology of the prepared composites was controlled by changing the amount of citrate and precipitation time. The morphologies of NiCo–PBA precursor and the 3D cubic hollow core–shell NiCo_2_O_4_@C composites are investigated by SEM and TEM measurements, which are shown in Figs. 1 and S1–S5. The NiCo–PBA precursor in Fig. 1b shows a uniform cube structure with an average size of approximately 400 nm. During the following heat treatment process, with the increasing temperature, the decomposition of –CN– is occurred, and the precursor cube undergoes a series of changes, the individual faces gradually depressed (Fig. 1c, d), forming a 3D NiCo_2_O_4_@C hollow core–shell microcubes framework (Fig. 1e, f), and eventually, the structure collapses (Fig. 1g). The NiCo_2_O_4_@C composites exhibit uniformly hollow core–shell microcubes structure, which inherited the size of NiCo–PBA precursor. The TEM is carried out to observe the microstructure of NiCo_2_O_4_@C composites. Figure 1h, i further identifies the hollow core–shell framework of the prepared composites. The lattice fringe spacings of 0.47, 0.24, and 0.25 nm in Fig. 1j are labeled as (111), (222), and (311) crystal planes of NiCo_2_O_4_. Furthermore, the TEM-based (Fig. 1k) and SEM-based EDS mapping result (Fig. S6) shows the uniform dispersion of Ni, Co, C, O, and N element in the NCO-3 composites. The results illustrate the transition of the NiCo–PBA precursor to NiCo_2_O_4_.

**Fig. 2:**
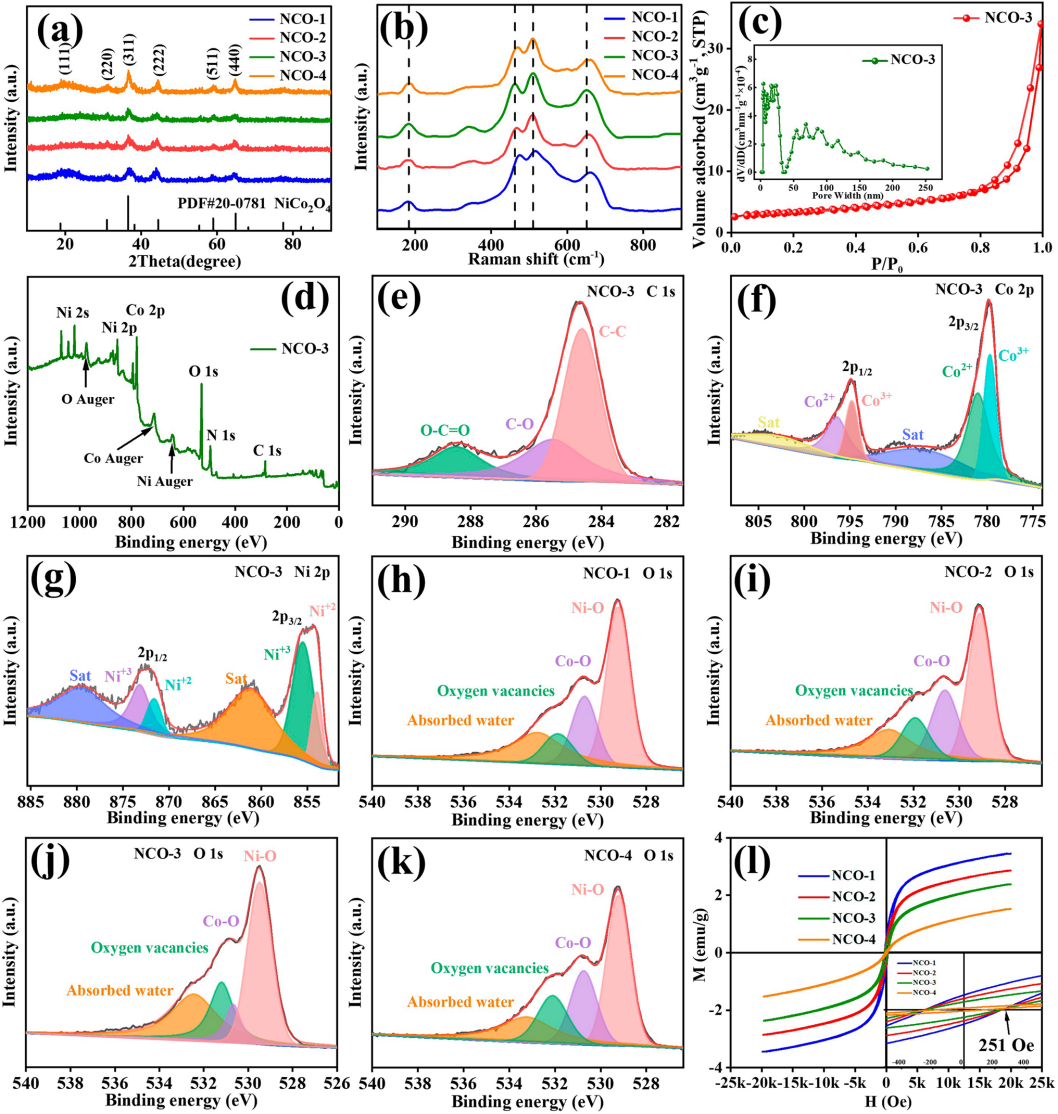
**a** XRD patterns, **b** Raman test of the prepared NCO; **c** Nitrogen adsorption/desorption isotherms and the pore size distributions, **d** XPS spectra of NCO-3, high-resolution XPS spectra for **e** C 1*s*, **f**) Co 2*p* and **g**) Ni 2*p* of NCO-3; XPS spectra of O 1*s* of **h** NCO-1, **i** NCO-2, **j** NCO-3 and **k** NCO-4; **l** the magnetization curve of NCO

The phase structure and crystallinity of precursors and NCO is characterized by XRD (Figs. 2a and S7). The Ni–Co PBA precursors in Fig. S1 is detected as Ni_3_[Co(CN)_6_]_2_·12H_2_O (JCPDS NO. 89–3738). Then the Ni–Co PBA precursors transform into NiCo_2_O_4_/C hybrids_,_ where the obvious diffraction peaks located at the 2θ of 18.91°, 31.15°, 36.70°, 44.62°, 59.10° and 64.98° in Fig. 2a are attributed to the (111), (220), (311), (400), (511), and (440) planes of NiCo_2_O_4_ (PDF #NO.20–0781) [20]. These results further prove the successful preparation of NiCo_2_O_4_/C microcubes.

Figure 2b shows the Raman spectra of the prepared composite, where the four distinct stretching vibration peaks at 184, 463, 510, and 651 cm^−1^ are detected as F_2g_, E_g_, F_2g_, and A_1g_ signals of NiCo_2_O_4_ orderly [21, 22], which further prove the formation of NiCo_2_O_4_. The peaks at ~ 510 and ~ 651 cm^−1^ are in connection with the vibration of oxygen ions (tetrahedral) and Co–O (octahedral), separately. The differences in F_2g_ and A_1g_ peak positions for the four samples, where 517 and 660 cm^−1^ for NCO-1, 509 and 656 cm^−1^ for NCO-2 and 509 and 651 cm^−1^ for NCO-4, is attribute to the disruption of lattice symmetry by defects caused during the calcination process. The peaks move towards lower frequencies with the increase of heat treatment temperature that are inferred to promote the generation of more oxygen vacancy defects, which induces defect polarization to enhance the electromagnetic absorption property [23]. To explore the detailed microstructure characteristics of the prepared composites, the N_2_ adsorption/desorption isotherms and pore size distribution curve of NCO-3 are displayed in Fig. 2c. The typical type IV isotherms can be observed directly, indicating the presence of mesoporous structure [24–26]. The specific surface area of NCO-3 is calculated to be *ca.* 11.2 m^2^ g^−1^, and the pore size mainly distributes between 1 and 150 nm.

The valence state and chemical composition of the prepared NiCo_2_O_4_/C hybrids is explored by the XPS test (Fig. 2d–k). The XPS spectra of NCO-3 illustrate the existing elements of C 1*s*, Co 2*p*, Ni 2*p*, and O 1*s*, which further confirm the successful synthesis of NiCo_2_O_4_/C composites. The three split peaks in the spectra of C 1*s* in Fig. 2e are attributed to the C–C (284.6 eV)、C–O (285.6 eV), and O–C=O (288.5 eV), respectively [27]. The Co 2*p* spectra (Fig. 2f) can be fitted to two spin–orbit doublets characteristics of Co^2+^ (780.98 and 796.5 eV) and Co^3+^ (788 and 805 eV), accompanying two satellite peaks (788 and 805 eV) [28, 29]. Meanwhile, the Ni 2p emission spectra (Fig. 2g) can be fitted to two spin–orbit doublets features of Ni^2+^ and Ni^3+^, accompanying two shakeup satellites [18]. Similarly, the spectra of Ni 2p are composed of two satellite peaks (862 and 880 eV) and two spin–orbit doublets features of Ni^2+^ (854 and 872 eV) and Ni^3+^ (856 and 874 eV) [30]. The O 1* s* spectrum consists of four categories of oxygen species (Fig. 2h–k). The peaks at 529.5 and 531.2 eV accord to the Ni–O and Co–O bond, respectively [31]. Simultaneously, the peak at 530.7 eV is related to oxygen vacancy, illustrating the presence of oxygen vacancy defects, consistent with the electron paramagnetic resonance (EPR) test results (Fig. S8). In our previous study, it was been proved that the content of oxygen vacancy defects is positively correlated with the electromagnetic wave absorption performance [32]. Therefore, the relative oxygen vacancy content of the NiCo_2_O_4_/C hybrids was calculated and compared based on the proportion of the integral area in Table S1. The NCO-3 presents the highest content of oxygen vacancies, which is favorable to generate dipole polarizations, illustrating the content of oxygen vacancy defects increases by the heat treatment temperature, and also influenced by structural integrity of the composites. Besides, the peak in 532.5 eV is indexed to the adsorbed water on the material surface [33].

To further detect the existence of carbon matrix in the prepared composites, the TGA measurement of NCO-3 is displayed in Fig. S9. In the process of heating, the mass of the sample gradually decreases; the weight change may be caused by the evaporation of water, the combustion of carbon, the decomposition and oxidation of the –CN– bond that generates carbon and nitrogen oxide gas and escapes into the air, and the conversion of NiCo_2_O_4_ into NiO and Co_3_O_4_ after heating up to 500 °C. in which Co^2+^ is oxidized to Co^3+^, and Ni^3+^ and O_2_ molecules are reduced to Ni^2+^ and O^2−^ [34].

The static magnetic property of the NiCo_2_O_4_ is tested at room temperature and shown in Fig. 2l. The coercively values of the absorbers are all around 251 Oe, indicating the similar storage capacity of magnetic energy in this series of samples [35]. The saturation magnetization (Ms) value gradually decreases with increasing temperature, and the maximum Ms was only 3.45 emu g.^−1^ for the NCO-1, which illustrate the hysteresis loss with low saturation magnetization contributes little to the magnetic loss of the absorbers. [36]

EPR results in Fig. S8 prove the existence of oxygen vacancy (O_V_) [20], which depends on Eq. (1) [24]:

1 $$hv=g\beta B$$

where $h$ represents Planck’s constant, $v$ is frequency, $g$ means constant, $\beta$ means Bohr magneton, and $B$ mean applied magnetic field. The recognized $g$ value of materials with O_V_ is ca. 2.00, which is associated with the nature of radical. The $g$ value of the NiCo_2_O_4_/C hybrids is calculated to be 2.00477, 2.00466, 2.00497, and 2.00494, respectively, which further indicate the occurrence of oxygen vacancy. The oxygen vacancies act as point defects, which will trap electrons and disrupt the equilibrium distribution of charges, leading to polarization and loss of electromagnetic energy under the altering electric field.

**Fig. 3:**
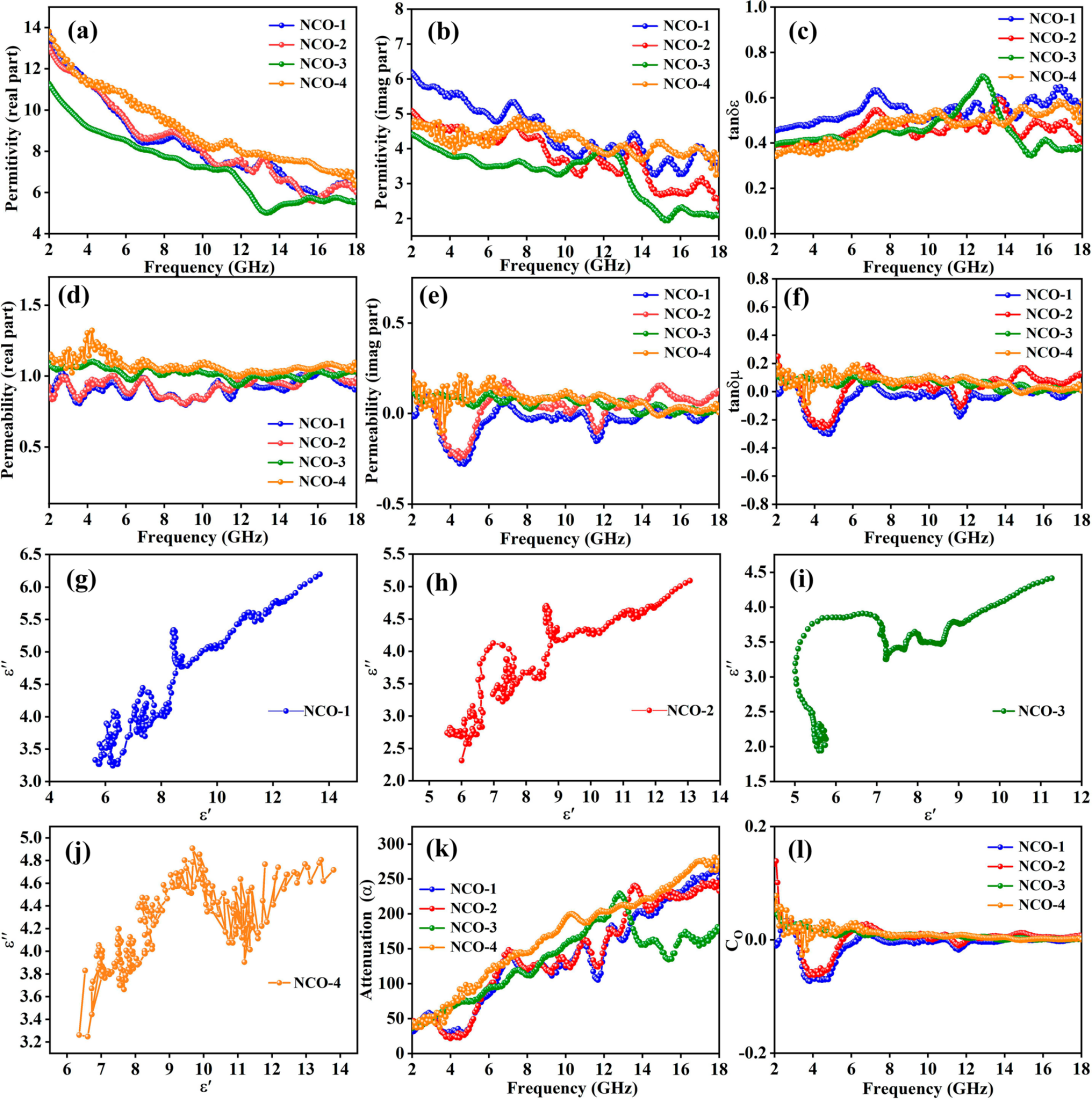
Frequency dependence of **a** $\varepsilon^{\prime }$ and **b** $\varepsilon^{\prime \prime }$; **c** tan $\delta \varepsilon$ and **d** $\mu^{\prime }$; **e** $\mu^{\prime \prime }$; **f**) tan $\delta \mu$; **g–j** Cole–Cole semicircles; **k** Attenuation constant $\alpha$, and **l** $C_{0}$ of prepared composites

The EMW absorbing properties of the synthesized NiCo_2_O_4_/C composites (with filling ratio of 40%) are dominated by the complex permittivity ($\varepsilon_{r}$ = $\varepsilon^{\prime }$ − $j\varepsilon^{\prime \prime }$) and permeability ($\mu_{r}$ = $\mu^{\prime }$ − $j\mu^{\prime \prime }$). The electromagnetic parameters of the absorbers are obtained from the vector network analyzer test that shown in Fig. 3. The real parts of permittivity ($\varepsilon^{\prime }$), decreases accompanied by the increasing frequency, and some fluctuations are observed in the curve (Fig. 3a). The imaginary part ($\varepsilon^{\prime \prime }$) of the complex permittivity (Fig. 3b) and dielectric loss tangent (Fig. 3c) presents multiple vibrational peaks from 6.5 to 18 GHz, indicating the existence of Debye relaxation, which corresponds well with the results of Cole–Cole semicircles (Fig. 3g–j). The dipole polarization formed in the prepared absorbers due to the abundant oxygen vacancies. And interfacial polarization between heterogeneous interfaces (NiCo_2_O_4_, carbon, and air) is inferred to contribute to satisfactory properties.

The complex permeability of the absorbers fluctuates in a low-value range (0.79 to 1.32 and −0.28 to 0.21, respectively) for both $\mu^{\prime }$ and $\mu^{\prime \prime }$ (Fig. 3d, e), which indicates a relatively poor magnetic loss capability [6]. The multiple resonance peaks (Fig. 3f) mainly originate from exchange resonance and natural resonance. Besides, eddy current loss is also an essential magnetic loss mechanism, which is described by the eddy current coefficient $(C_{0} = \mu^{\prime \prime } \left( {\mu^{\prime } } \right)^{2} f^{ - 1} )$ [37]. As shown in Fig. 3l, the $C_{0}$ of the NiCo_2_O_4_/C absorbers is almost a constant in the range of 6 to 18 GHz, suggesting a nonnegligible role of eddy current loss in the magnetic loss behavior.

The *RL* values are calculated according to transmission line theory, as shown in Eqs. 2 and 3 [38]:

2 $${Z}_{in}={Z}_{0}\sqrt{\frac{{\mu }_{r}}{{\varepsilon }_{r}}}\mathrm{tan}h\left|j\left(\frac{2\pi fd}{c}\right)\sqrt{{\mu }_{r}{\varepsilon }_{r}}\right|$$

3 $$RL=20lg\left|\frac{{Z}_{0}-{Z}_{in}}{{Z}_{0}+{Z}_{in}}\right|$$

where Z_in_ means the input impedance of the absorber, Z_0_ means the impedance of free space, ${\varepsilon }_{r}$ and ${\mu }_{r}$ represent the complex permittivity and complex permeability, respectively, $f$ means the frequency of the incident electromagnetic wave, $d$ is the thickness and c is the speed of the EMW. The *RL* value less than −10 dB illustrates more than ninety percent of the incident EMW could be absorbed and the corresponding frequency range with *RL* lower than −10 dB is considered as EAB [22].

**Fig. 4:**
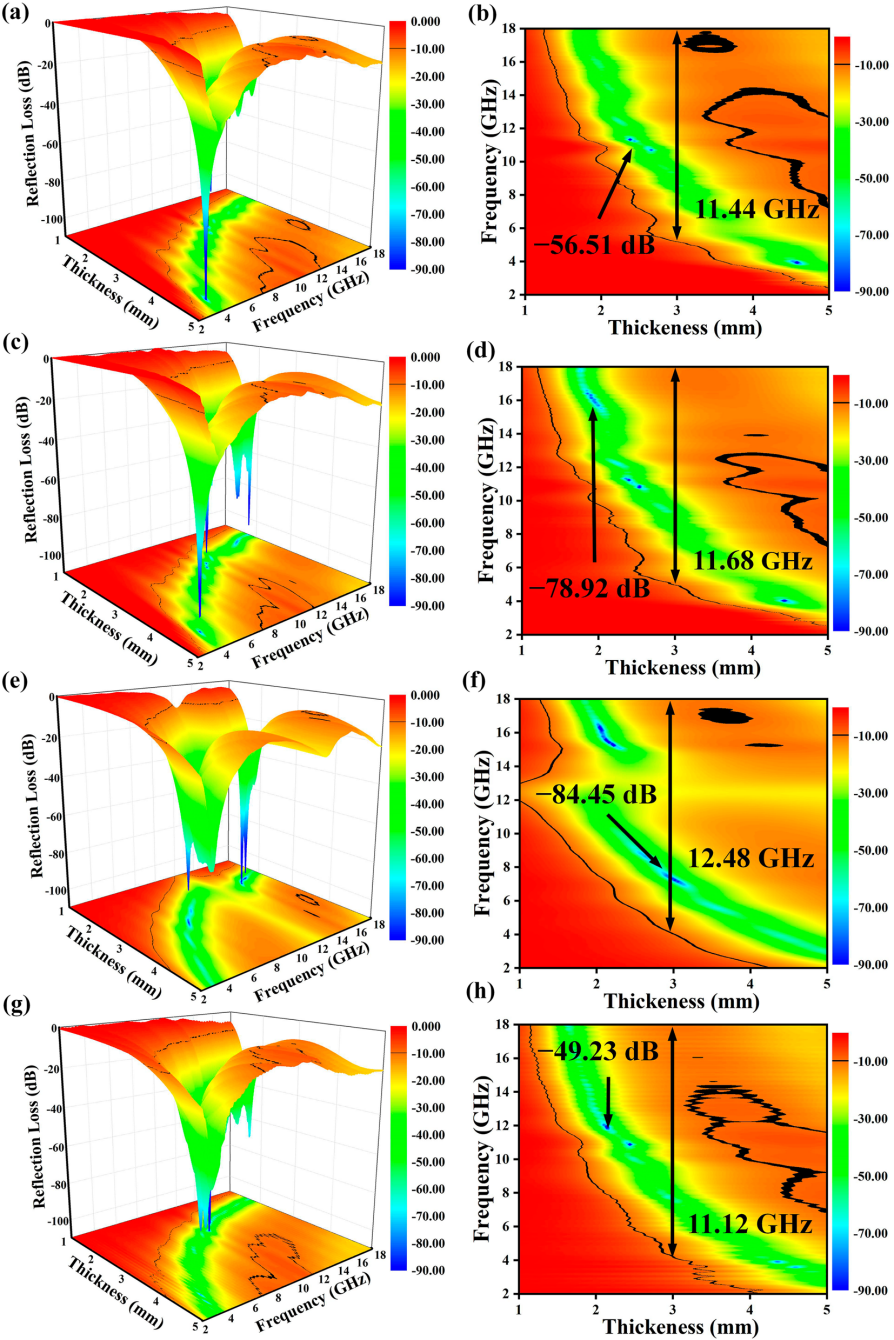
3D, 2D color map at different matching thicknesses of **a, b** NCO-1, **c, d** NCO-2, **e, f** NCO-3and **g, h** NCO-4

**Fig. 5:**
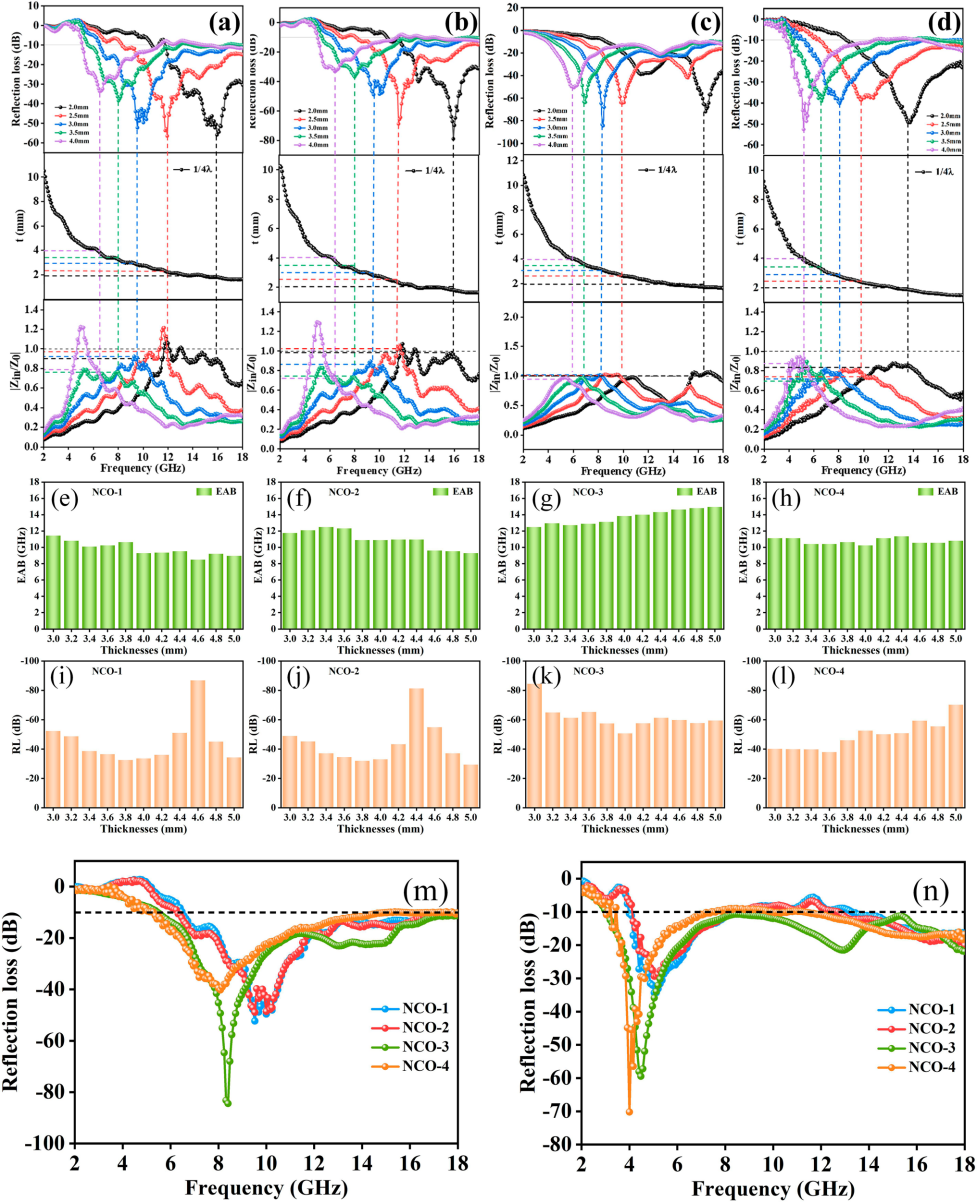
*RL* values vs. frequency at different thicknesses, the simulations of the absorber thickness vs. peak frequency under l/4 conditions, and the impedance matching characteristic for **a** NCO-1, **b** NCO-2, **c** NCO-3, and **d** NCO-4. Comparison of **e–h** EAB values and **i–l** *RL*_min_ at a matched thickness of 3–5 mm. Comparison of EMW absorption performance at a thickness of **m** 3 mm and **n** 5 mm

The EMW absorption performance of the as-synthesized NiCo_2_O_4_@C microcubes is presented in Figs. 4 and 5. The results illustrate NiCo_2_O_4_@C microcubes present outstanding EMW absorption performance in terms of the calculated *RL* and EAB values. An EAB value of 11.44 GHz for NCO-1 is obtained at 3.0 mm and the *RL*_min_ value reaches −56.51 dB at the thickness of 2.5 mm (Figs. 4a, b and 5a). For NCO-2, the EAB value increases to 11.68 GHz at 3.0 mm, and the *RL*_min_ value of −78.92 dB is obtained at 2.0 mm (Figs. 4c, d and 5b). A satisfactory EAB value of 11.12 GHz obtained at 3.0 mm and *RL*_min_ value of −49.23 dB at 2.0 mm are obtained for NCO-4, respectively (Figs. 4g, h and 5d). Encouragingly, an ultra-large EAB value as broad as 12.48 GHz (5.52 to 18 GHz) and *RL*_min_ value of −84.45 dB at the thickness of 3.0 mm (Figs. 4e, f and 5c), which is the largest among the NiCo_2_O_4_-based absorbers (Table 1). It is worth noting that none *RL* value for EMW absorbers at multiple matched thicknesses beyond −10 dB at 18 GHz, indicating that the absorbers display potential absorption capability in the higher frequency range. The absorption performance of the four EMW absorbers is compared in more detail by reducing the matching thickness from 0.5 to 0.2 mm, as shown in Fig. 5e–l, where the EAB of all the four EMW absorbers exceed 10 GHz and *RL*_min_ values exceed −20 dB (losing 99% of the incident electromagnetic wave) at these matched thicknesses. Figure 5m, n shows the comparison of EMW Absorption performance for all absorbers at the thickness of 3 and 5 mm, respectively, which further prove the NCO-3 possessed the optimum EAB value. Meanwhile, the EAB value even reaches 14.96 GHz at the thickness of 5.0 mm.

**Table 1:** Comparison of EMW absorbing properties of NiCo_2_O_4_-based absorbers

Absorbers	*RL*_min_ (dB)	EAB (GHz)	References
NiCo_2_O_4_	−32.56	5.81	[1]
C/NiCo_2_O_4_/ZnO	−43.61	4.32	[13]
NiCo_2_O_4_	−37.00	4.64	[14]
Co_3_O_4_@ NiCo_2_O_4_	−34.42	4.88	[15]
NiCo_2_O_4_	−42.80	6.08	[18]
NiCo_2_O_4_	−38.9	6.26	[20]
NiO/ NiCo_2_O_4_	−48.10	5.84	[23]
NiCo_2_O_4_	−47.01	2.98	[24]
C@NiCo_2_O_4_	−39	4.16	[30]
NiCo_2_O_4_/Co_3_O_4_/NiO	−28.63	4.72	[31]
NiCo_2_O_4_/HCNT	−55.9	4.8	[33]
NiO/ NiCo_2_O_4_	−57.40	6.08	[36]
MWCNT@ NiCo_2_O_4_	−41.5	5.0	[46]
NiCo_2_O_4_	−49.78	7.10	[47]
NiCo_2_O_4_@C	−84.45	12.48	This work

In order to investigate the attenuation mechanism for the excellent EMW absorption, the attenuation constant (α) is evaluated using Eq. 4 [39]:

4 $$\alpha = \frac{\sqrt 2 \pi f}{c} \times \sqrt {\mu^{\prime \prime } \varepsilon^{\prime \prime } - \mu^{\prime } \varepsilon^{\prime } + \sqrt {\left( {\mu^{\prime } \varepsilon^{\prime \prime } + \mu^{\prime \prime } \varepsilon^{\prime } } \right)^{2} + \left( {\mu^{\prime \prime } \varepsilon^{\prime \prime } - \mu^{\prime } \varepsilon^{\prime } } \right)^{2} } }$$

Figure 3k displays the α-values of the four prepared absorbers, which shows the result of NCO-4 > NCO-2 > NCO-1 > NCO-3. Theoretically, NCO-4 should present the optimum EMW absorption capacity, but the impedance matching factor should be considered.

For exploring deeper into the cause of the excellent EM wave dissipation behavior of the NiCo_2_O_4_/C absorbers, the impedance matching characteristic ($Z= |{Z}_{in}/{Z}_{0}|$) is computed and exhibited in Figs. 4 and 5. Typically, the better the impedance matching for the absorber [40], the less reflection the incident EMW. The results show that the impedance matching of the prepared absorbers floats around 1.0 at a suitable matching thickness [41]. By further comparative analysis, NCO-3 displays the most outstanding impedance match. Based on the synergistic effect of impedance matching and attenuation constants, the NCO-3 presents a superior EMW absorption capability.

Furthermore, EMW absorption capability of NCO-3 with a filling ratio of 20% and 30% was tested, which are presented in Figs. S10–S12. The NCO-3 with filling ratio of 20% exhibits an EAB of 5.44 GHz at 3.0 mm and an *RL*_min_ value of −20.44 dB at 3.5 mm and NCO-3 with filling ratio of 30% exhibits an EAB value of 4.96 GHz at 2.5 mm and an *RL*_min_ value of −46.02 dB at 5.0 mm, respectively, which demonstrates noteworthy impact of the filling ratio for EMW absorption capability.

For better understanding of the outstanding EMW absorption performance of the NiCo_2_O_4_/C absorber, the quarter wavelength theory was applied by Eq. 5 [42]:

5 $$t_{m} = \frac{n\lambda }{4} = \frac{nc}{{4f_{m} \sqrt {\left| {\varepsilon_{r} } \right|\left| {\mu_{r} } \right|} }} \left( {n = 1, 3, 5...} \right)$$

where $t_{m}$ and $f_{m}$ mean the optimal thickness and frequency of the absorbers. The matching results of *RL*_min_, thickness, and frequency are shown in Fig. 5. The intersection of the dashed line and the t-curve indicate the matching thickness vs. the peak frequency collected in Fig. 5a–d. The positions of these data are around the 1/4 λ, indicating these absorbers are in accordance with the quarter wavelength theory. Therefore, incident and reflected EMW would be canceled by each other in the interface of air-absorbers as the phase difference of 180°, maximizing the EMW attenuation ability.

**Fig. 6:**
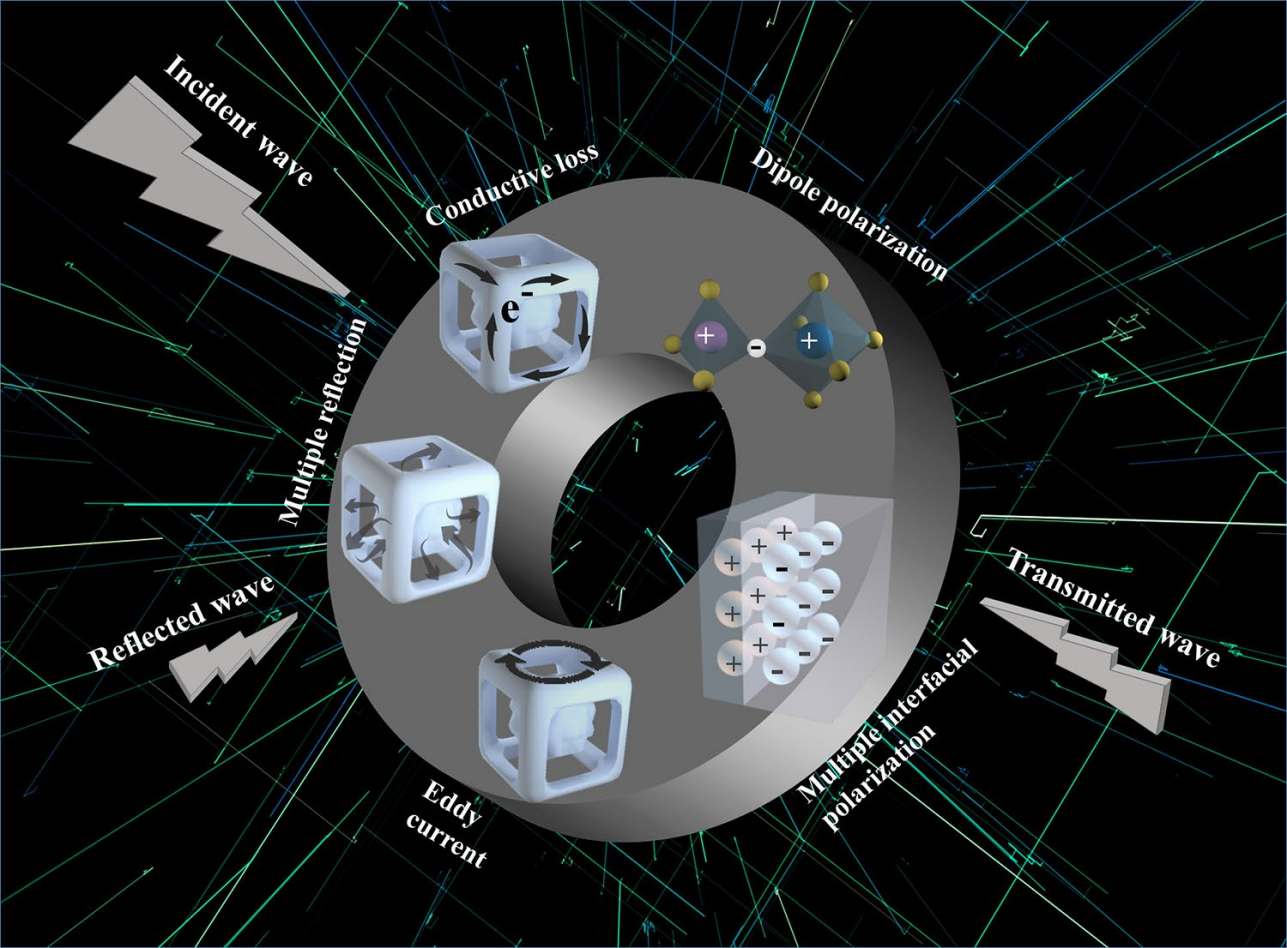
Schematic illustration of EMW absorption mechanism

The outstanding EMW dissipation of NCO-3 with an ultrabroad EAB value of 12.48 GHz is achieved, and the attenuation mechanisms are summarized in Fig. 6. The outstanding impedance matching characteristics allow EMW to enter the absorbers to the greatest extent firstly. Secondly, these controllable oxygen vacancy defects act as dipoles and induce dipole polarization. The dipoles would change their arrangement from disorder to order when an electric field is applied, and the incident EMW will be effectively dissipated during this period [43]. Thirdly, the existence of eddy current loss and interface polarization induced by the accumulation of different charges at the heterogeneous interfaces, are beneficial to the loss effect of EMW. Fourthly, the different binding energy for electric charges on the sides of the interface leads to differences in the amount of charge migration in and out of electromagnetic fields, resulting in interface polarization. Finally, when the EMW enters the inside framework, multiple reflections between the gaps are happened to increase the propagation path and then dissipate the EM waves [44, 45]. Furthermore, macroscopic current would be generated in the absorber under an applied electromagnetic field, and the EMW would be converted into heat energy based on the Joule theory.

## Conclusions

In summary, the 3D core–shell NiCo_2_O_4_@C hollow microcubes are synthesized by the environmentally-friendly sacrificial template method. The oxygen vacancy defects are introduced into the special framework due to the sensitive response of NiCo_2_O_4_ to temperature changes. The evolution of morphology with temperature affected the density, distribution of heterogeneous interfaces, and the content of oxygen vacancy defects of the prepared composites, thus affecting the impedance matching and attenuation capability of the absorber. Benefiting from the synergistic EMW absorption mechanisms, including multiple reflections and scattering induced by the abundant pores, interfacial polarization induced by multiple components, dipole polarization induced by the defects, conductive loss, and eddy current loss, the optimized NCO-3 presented ultrabroad EAB value of 12.48 GHz (5.52 to 18 GHz), and *RL*_min_ value of −84.45 dB at 8.4 GHz, covering the entire X and Ku band and even extends to the K band, which is the broadest among the reported NiCo_2_O_4_-based absorbers. It is believed that this report could illuminate the route for applications of high-performance NiCo_2_O_4_-based and other transition metal oxides-based EMW absorbers.

## Supplementary Information

Below is the link to the electronic supplementary material.

Supplementary file1 (PDF 943 KB)
