# Correction: Initiating Binary Metal Oxides Microcubes Electromagnetic Wave Absorber Toward Ultrabroad Absorption Bandwidth Through Interfacial and Defects Modulation

**DOI:** 10.1007/s40820-025-01785-2

**Published:** 2025-05-30

**Authors:** Fushan Li, Nannan Wu, Hideo Kimura, Yuan Wang, Ben Bin Xu, Ding Wang, Yifan Li, Hassan Algadi, Zhanhu Guo, Wei Du, Chuanxin Hou

**Affiliations:** 1 School of Environmental and Material Engineering, Yantai University, No. 30 Qingquan Road, Yantai, 264005 Shandong People’s Republic of China; 2 School of Material Science and Engineering, Shandong University of Science and Technology, 266590 Qingdao, People’s Republic of China; 3 Mechanical and Construction Engineering, Faculty of Engineering and Environment, Northumbria University, Newcastle Upon Tyne, NE1 8ST UK; 4 College of Materials Science and Engineering, Taiyuan University of Science and Technology, Taiyuan, 030024 People’s Republic of China; 5 Department of Electrical Engineering, Faculty of Engineering, Najran University, 11001 Najran, Saudi Arabia

**Correction to: Nano-Micro Letters (2023) 15:220** 10.1007/s40820-023-01197-0

Following publication of the original article [1], the authors found that they pasted the same data when drawing XRD for sample NCO-1 and NCO-2 in Fig. 2a, however, the XRD of all four samples in the manuscript was tested, and XRD raw data were kept and can be offered.

The correct Fig. 2 has been provided in this Correction.

The incorrect Fig. 2 is:
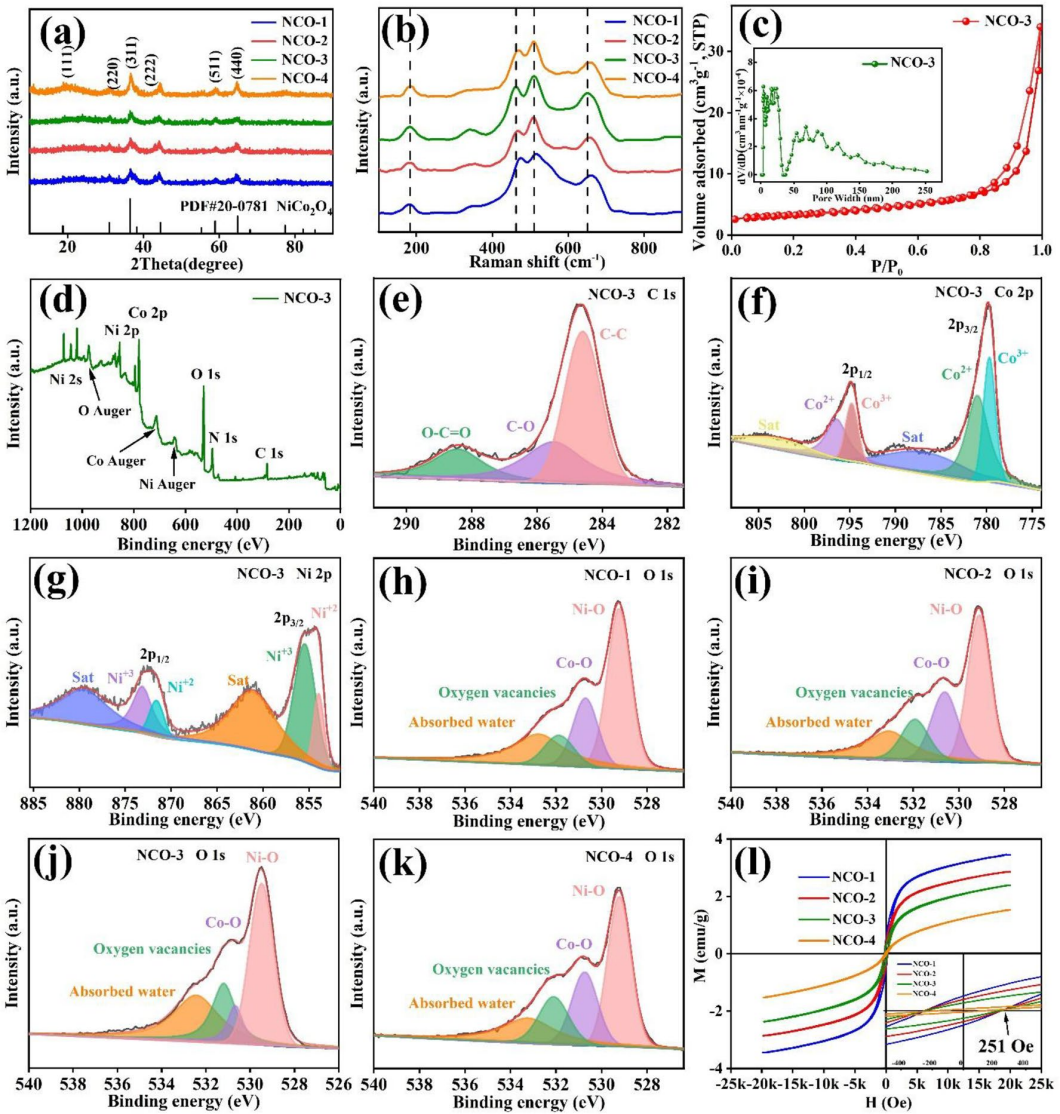


**Fig. 2 a** XRD patterns, **b** Raman test of the prepared NCO; **c** Nitrogen adsorption/desorption isotherms and the pore size distributions, **d** XPS spectra of NCO-3, high-resolution XPS spectra for **e** C 1*s*, **f**) Co 2*p* and **g**) Ni 2*p* of NCO-3; XPS spectra of O 1*s* of **h** NCO-1, **i** NCO-2, **j** NCO-3 and **k** NCO-4; **l** the magnetization curve of NCO

The correct Fig. 2 is:
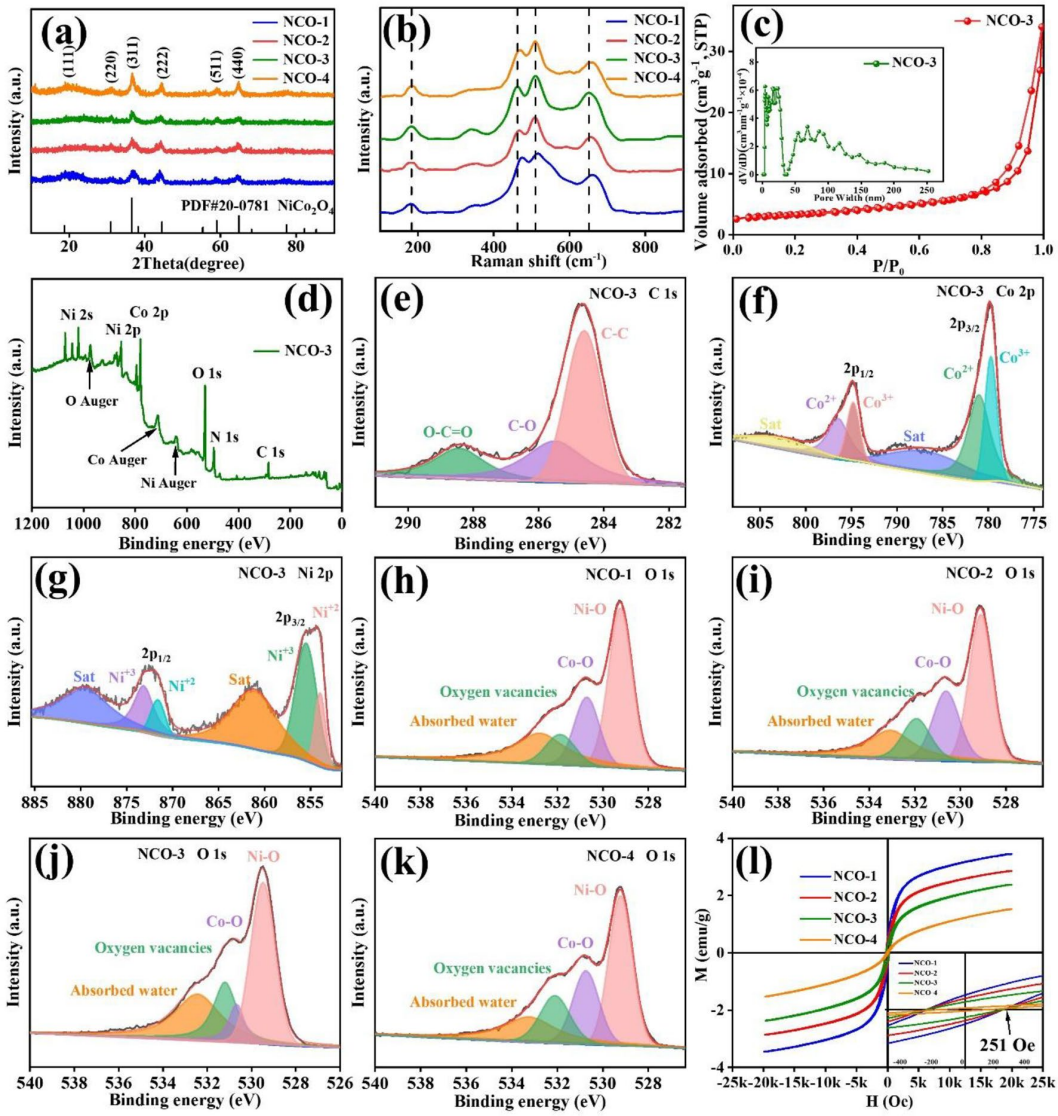


**Fig. 2 a** XRD patterns, **b** Raman test of the prepared NCO; **c** Nitrogen adsorption/desorption isotherms and the pore size distributions, **d** XPS spectra of NCO-3, high-resolution XPS spectra for **e** C 1*s*, **f** Co 2*p* and **g** Ni 2*p* of NCO-3; XPS spectra of O 1*s* of **h** NCO-1, **i** NCO-2, **j** NCO-3 and **k** NCO-4; **l** the magnetization curve of NCO

The original article [1] has been corrected.
