# Supplementary material for: Initiating Binary Metal Oxides Microcubes Electromagnetic Wave Absorber Toward Ultrabroad Absorption Bandwidth Through Interfacial and Defects Modulation
Source: Nanomicro Lett. 2023 Oct 9;15:220. doi: 10.1007/s40820-023-01197-0 (PMC10562357; doi:10.1007/s40820-023-01197-0)
Supplement: Supplementary file 1 — Supplementary file1 (PDF 943 KB) [file 40820_2023_1197_MOESM1_ESM.pdf]

Supporting Information for

Initiating Binary Metal Oxides Microcubes Electromagnetic Wave Absorber toward Ultrabroad Absorption Bandwidth through Interfacial and Defects Modulation

Fushan Li[1], Nanan Wu[2], Hideo Kimura[1], Yuan Wang[3], Ben Bin Xu[3,*], Ding Wang[3], Yifan Li[3], Hassan Algadi[4,5], Zhanhu Guo[3,*], Wei Du[1,*], and Chuanxin Hou[1,*]

[1]School of Environmental and Material Engineering, Yantai University, No. 30 Qingquan Road, Yantai, Shandong, 264005, P. R. China

[2]School of Material Science and Engineering, Shandong University of Science and Technology, Qingdao, 266590, P. R. China

[3]Mechanical and Construction Engineering, Faculty of Engineering and Environment, Northumbria University, Newcastle Upon Tyne, NE1 8ST, UK

[4]College of Materials Science and Engineering, Taiyuan University of Science and Technology, Taiyuan, 030024, P. R. China

[5]Department of Electrical Engineering, Faculty of Engineering, Najran University, Najran, 11001, Saudi Arabia

*Corresponding authors. E-mail: duwei@ytu.edu.cn (Wei Du), zguo10@utk.edu (Zhanhu Guo), ben.xu@northumbria.ac.uk (Ben Bin Xu), chuanxin210@ytu.edu.cn (Chuanxin Hou)

Supplementary Figures and Tables

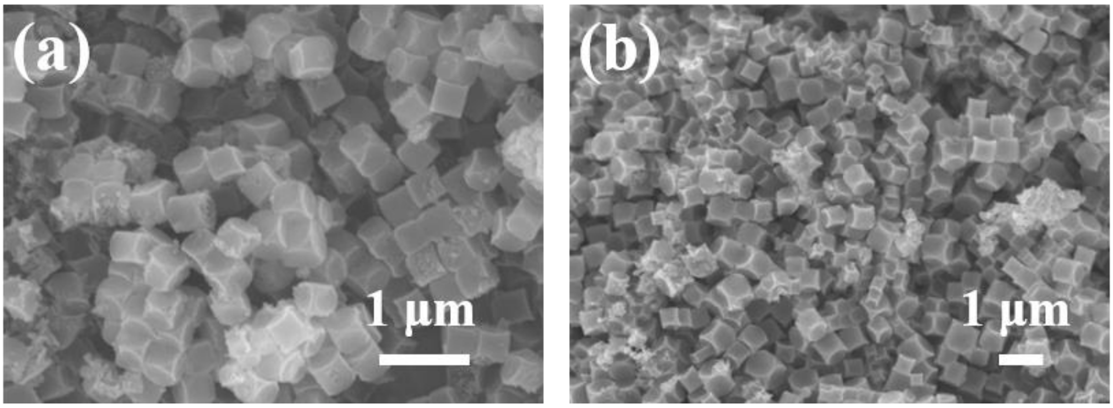

Fig. S1 SEM images of Ni-Co-PBA

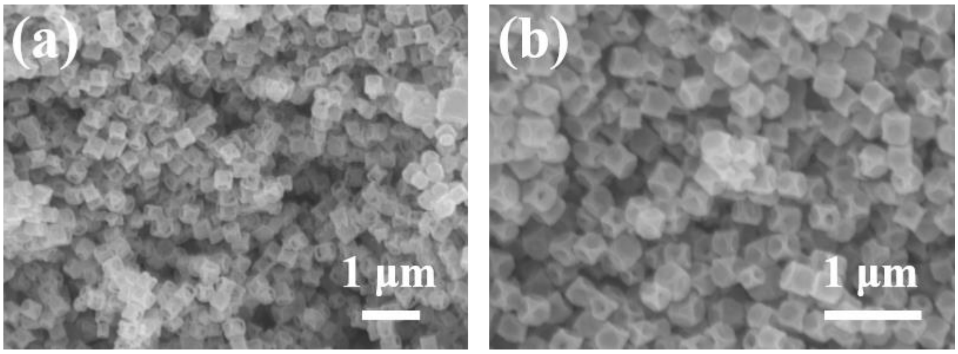

Fig. S2 SEM images of NCO-1

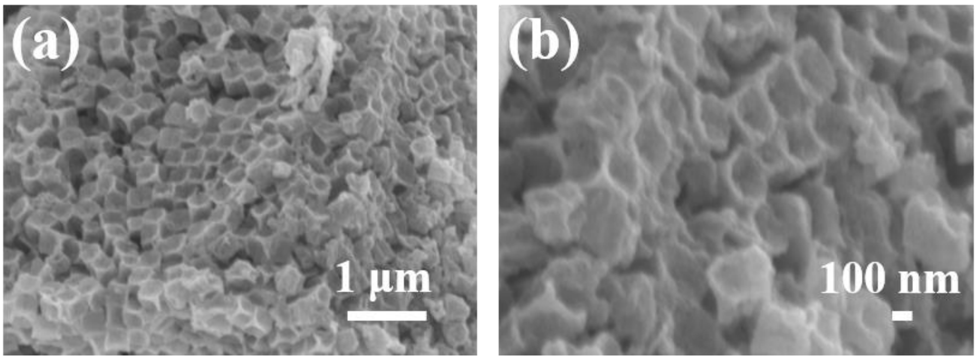

Fig. S3 SEM images of NCO-2

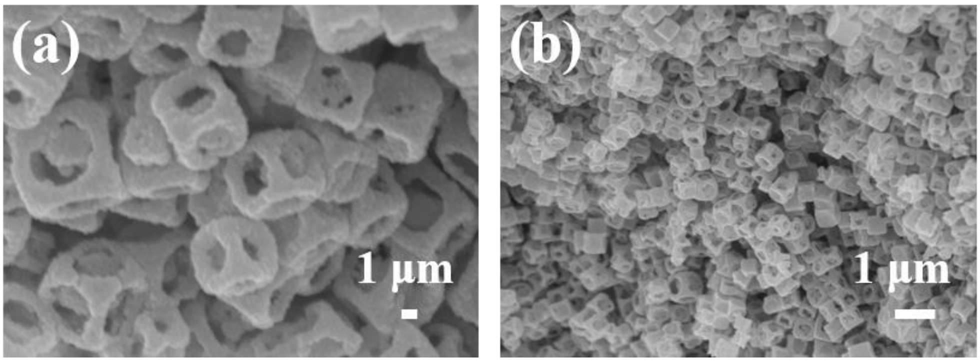

Fig. S4 SEM images of NCO-3

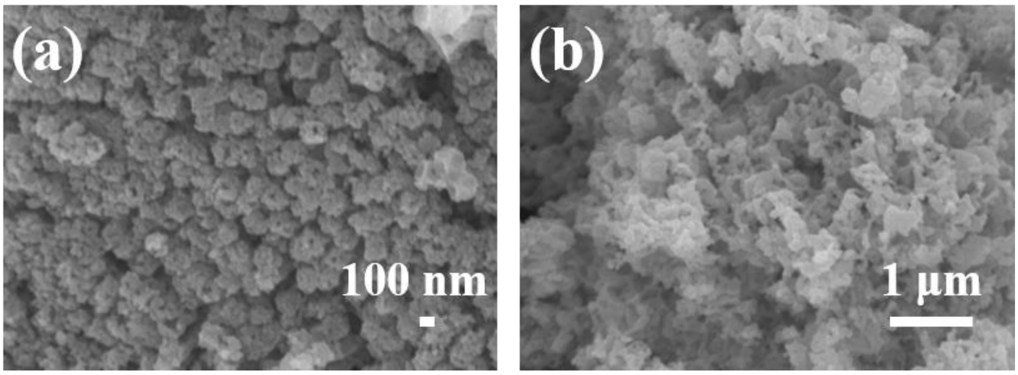

Fig. S5 SEM images of NCO-4

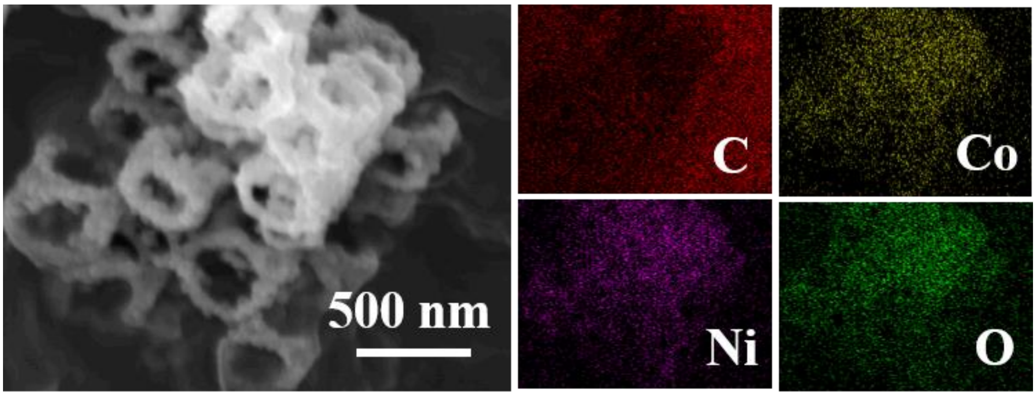

Fig. S6 EDS spectrum of NCO-3

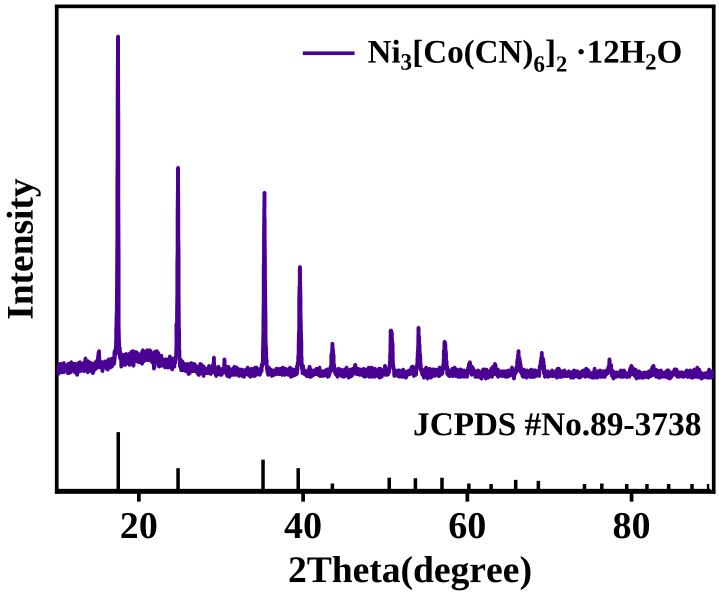

Fig. S7 XRD patterns of Ni-Co PB

Table S1 Comparison of relative oxygen vacancy levels

Sample	Oxygen vacancy	O 1s	Relative oxygen vacancy levels (%)
NCO-1	21226	205689	0.103195
NCO-2	27631	183178	0.150842
NCO-3	38109	193048	0.197407
NCO-4	29284	169477	0.17279

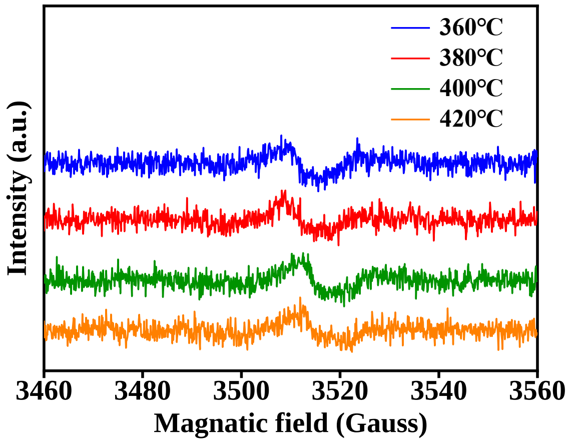

Fig. S8 Electron Paramagnetic Resonance curves of the samples

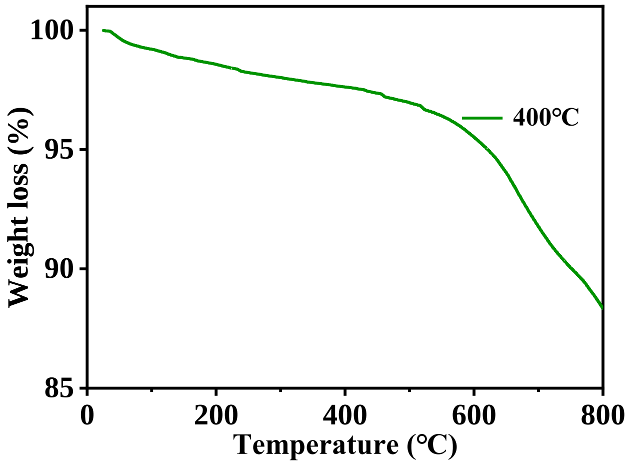

Fig. S9 TGA curves of NCO-3

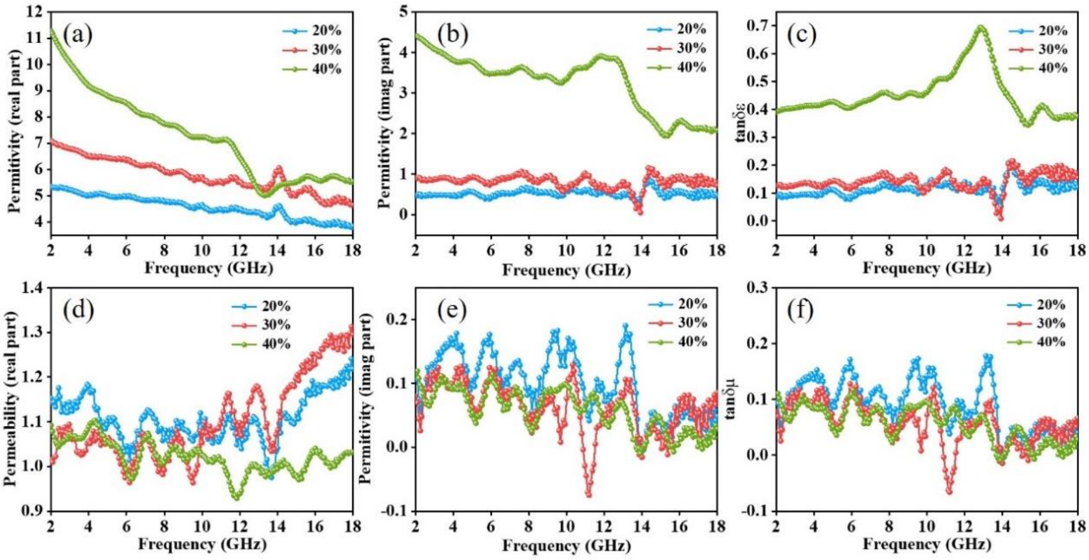

Fig. S10 Frequency dependence of **a**) ε' and **b**) ε''; **c**) $\tan\delta\varepsilon$ and **d**) μ'; e) μ''; **f**) $\tan\delta\mu$ of NCO-3 with different filling ratios

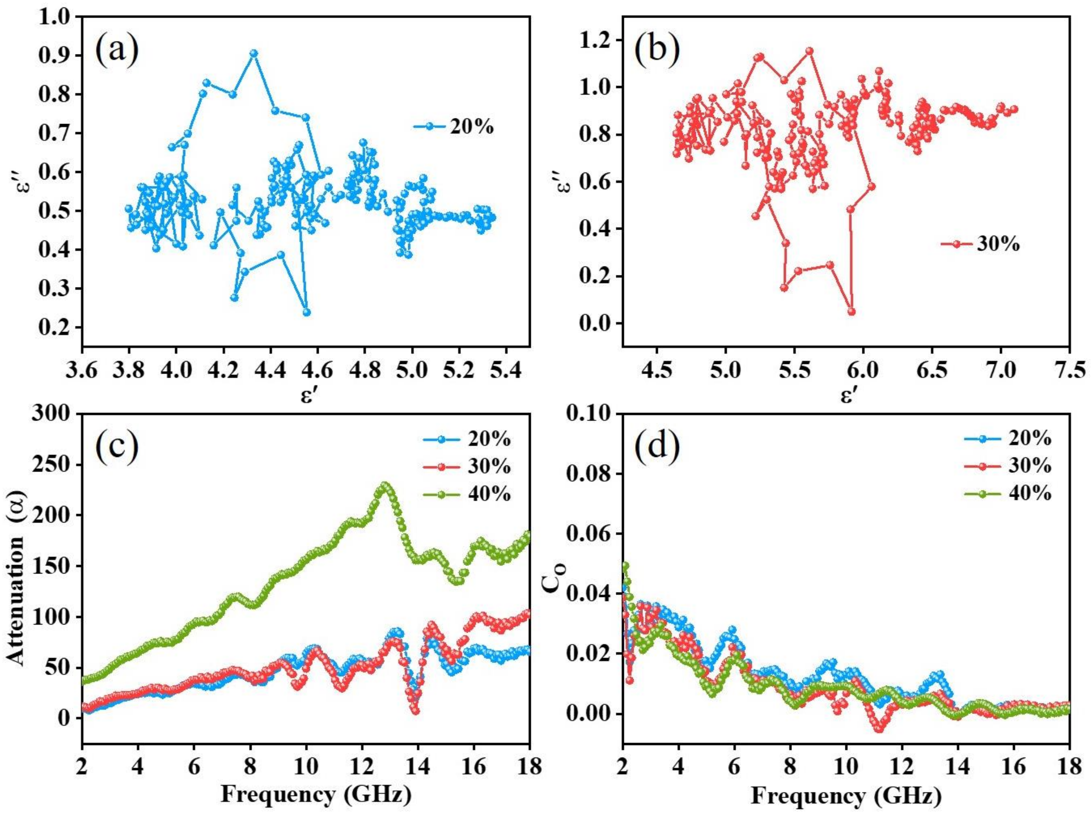

Fig. S11 a-b) Cole-Cole semicircles; **c**) Attenuation constant, and **d**) C_0 of NCO-3 with different filling ratios

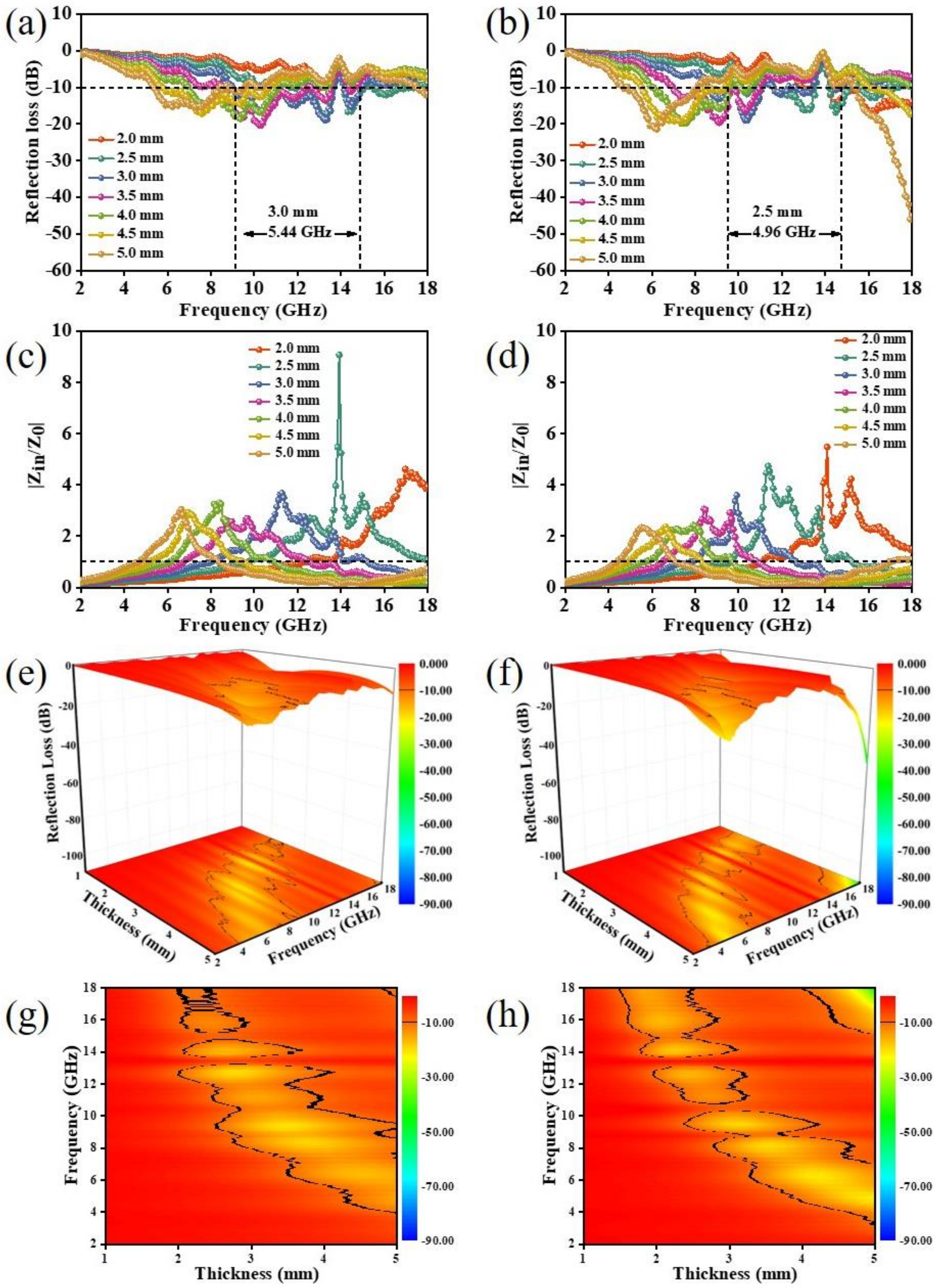

Fig. S12 RL value vs. frequency at different thickness, the impedance matching characteristic, 3D plots and 2D counter maps for **a, c, e, g**) NCO-3-20% and **b, d, f, h**) NCO-3-30%
